# Hemodynamic evaluation following ductal closure and association with IVH in premature infants born at 22–23weeks gestation

**DOI:** 10.3389/fped.2026.1825698

**Published:** 2026-06-12

**Authors:** Tomonori Kurimoto, Katsuaki Toyoshima, Hiroshi Ohashi, Jun Shibasaki, Tomoyuki Shimokaze, Tomoko Saito, Kaoru Katsumata, Asataro Yara, Eiji Hirakawa, Takuya Tokuhisa

**Affiliations:** 1 Department of Neonatology, Perinatal Medical Center, Kagoshima City Hospital, Kagoshima, Japan; 2 Department of Neonatology, Kanagawa Children’s Medical Center, Yokohama, Japan

**Keywords:** end-systolic wall stress, extremely preterm infants, intraventricular hemorrhage (IVH), mean velocity of circumferential fiber shortening, patent ductus arteriosus (PDA)

## Abstract

**Background:**

Intraventricular hemorrhage (IVH) remains a major complication in extremely preterm infants, but the immediate hemodynamic changes associated with hemorrhage after ductal closure are not well defined.

**Methods:**

This was a two-center retrospective cohort study conducted at two tertiary neonatal intensive care units. Hemodynamic parameters, including left ventricular diastolic diameter, mean velocity of circumferential fiber shortening, end-systolic wall stress, mean arterial pressure, heart rate, and internal cerebral vein waveform grade, were assessed serially from birth to 72 h of life. Generalized linear mixed-effects models were used to evaluate temporal associations between post-closure hemodynamic changes and IVH.

**Results:**

In this two-center retrospective cohort study, 26 infants born at 22–23 weeks’ gestation were included. Seven infants developed grade 2–4 IVH, all first detected after PDA closure, while 19 who did not IVH. Infants who developed IVH showed a distinct post-closure pattern characterized by increased end-systolic wall stress, mean arterial pressure, and heart rate, together with reduced mean velocity of circumferential fiber shortening, within 12 h after ductal closure. Between-group differences were significant at 6 and 12 h after closure (all *p* < 0.01).

**Conclusion:**

Hemodynamic instability emerging within 12 h after ductal closure was associated with subsequent IVH in infants born at 22–23 weeks’ gestation. These findings identify the immediate post-closure period as a critical window for circulatory monitoring and potential targeted intervention.

## Introduction

At birth, a significant decrease in pulmonary vascular resistance leads to a marked increase in pulmonary blood flow. This change imposes a volume load on the left ventricle due to increased pulmonary blood flow, along with a pressure load resulting from the cessation of placental circulation. During fetal life, the ductus arteriosus shunts blood from the pulmonary artery to the aorta. However, after birth, as pulmonary vascular resistance decreases, the direction of blood flow through the ductus arteriosus reverses from the aorta to the pulmonary artery, eventually leading to ductal closure.

Although ductal closure has often been regarded as a cause of increased left ventricular afterload and higher end-systolic wall stress (ESWS), this relationship is not straightforward. Because blood pressure also rises during the same period, it is difficult to attribute afterload elevation solely to ductal closure (1). Moreover, some infants show a reduction in left ventricular dimension after patent ductus arteriosus (PDA) closure, leading to decreased wall stress, while in others—whose ventricular size does not decrease despite reduced preload—ESWS may increase (2, 3).

These hemodynamic changes present a considerable challenge for extremely preterm infants to adapt to (4–6). Previous studies have reported that preterm infants often experience impaired cardiac function between 4 and 76 h of life, leading to reduced systemic perfusion and cerebral blood flow, which may subsequently result in intraventricular hemorrhage (IVH) (7). Approximately 90% of IVH cases occur within the first 72 h after birth (8). Echocardiographic parameters used in neonatal intensive care units (NICUs) to assess cardiac function include the mean velocity of circumferential fiber shortening corrected for heart rate (mVcfc), which indicates left ventricular contractility; the left ventricular end-diastolic diameter (LVDd), representing preload and the left ventricular ESWS, reflecting afterload (9–11). Although most IVH occurs within the first 72 h after birth, the immediate hemodynamic response following ductal closure in infants born at 22–23 weeks’ gestation has not been well characterized. We therefore aimed to examine whether early hemodynamic changes within 12 h after PDA closure were associated with subsequent grade 2–4 intraventricular hemorrhage in this population.

## Materials and methods

### Study design and participants

This was a two-center retrospective cohort study conducted at two tertiary neonatal intensive care units (Kagoshima City Hospital and Kanagawa Children’s Medical Center). The study included neonates born at 22 weeks and 0 days to 23 weeks and 6 days of gestation. Gestational age was determined based on the last menstrual period, early pregnancy ultrasound measurements (crown–rump length), or both.

Neonates with birth weights below the 10th percentile based on standardized Japanese reference data were classified as having fetal growth restriction (FGR).

The patients were actively resuscitated and admitted to the NICU. Active resuscitation included continuous positive airway pressure, endotracheal intubation, and surfactant replacement therapy, and was provided to all neonates. Data were collected from the electronic medical records.

Inclusion criteria were neonates born within the specified gestational age range who underwent serial hemodynamic assessment during the first 72 h of life.

Exclusion criteria included neonates with intraventricular hemorrhage detected on the initial cranial ultrasound examination at admission.

IVH was defined according to the Volpe classification (grade 2–4) (12).

This study was approved by the institutional review boards of the participating institutions, and the requirement for informed consent was waived because of the retrospective design.

### Management and outcomes

The antenatal corticosteroid (ACS) course was defined as the administration of at least two doses of ACS before delivery during the current pregnancy to promote fetal lung maturation (13). The pathological placental findings included chorioamnionitis and funisitis, staged according to the Blanc classification (14). Bacteremia was defined as the detection of microorganisms in blood cultures. Cranial ultrasonography and echocardiography were performed by attending neonatologists three to four times during the first four postnatal days. Cranial ultrasonography was used to evaluate IVH and internal cerebral vein (ICV) pulsation and echocardiography was used to measure LVDd, mVcfc and ESWS at each examination. ICV pulsation was semi-quantitatively graded based on waveform characteristics, as previously described (15). Briefly, grade 0 represents continuous non-pulsatile flow; grade 1, mild pulsatility with a minimum-to-maximum velocity ratio ≥ 0.5; grade 2, pronounced pulsatility with a ratio < 0.5; and grade 3, intermittent or absent flow with a minimum velocity approaching zero. Echocardiography was performed at each examination using standard parasternal short-axis and long-axis views. Left ventricular internal dimensions at end-diastole (LVIDd) and end-systole (LVIDs) were obtained from M-mode recordings at the level of the papillary muscles. From these measurements, left ventricular systolic function indices were calculated. The mean velocity of circumferential fiber shortening corrected for heart rate (mVcfc) was calculated as: mVcfc (circ/s) = {(LVIDd−LVIDs)/LVIDd} × RR^1/2^/ET.

End-systolic wall stress (ESWS) was calculated using the following formula: ESWS (g/cm²) = 1.35 × (LVIDs×Pes)/{4 × Hes×(1 + Hes/LVIDs)}.

Where LVIDd is the left ventricular internal dimension at end-diastole, LVIDs is the left ventricular internal dimension at end-systole, Pes is the end-systolic pressure, Hes is the posterior wall thickness at end-systole, ET is the left ventricular ejection time, and RR is the RR interval. End-systolic pressure (Pes) was estimated from arterial blood pressure waveforms by assigning systolic pressure to the peak of the trace and diastolic pressure to the nadir, with linear interpolation to the level of the dicrotic notch (9). Additional measurements included left atrial (LA) and aortic root diameters obtained from the parasternal long-axis view using the leading-edge approach. LA area and longitudinal length were determined from the apical four-chamber view, and left atrial volume was estimated using a single-plane area–length method based on a previously published equation. PDA was evaluated primarily in the ductal long-axis view. The smallest internal ductal diameter (PDAd, mm) was identified in this view. Flow direction across the ductus (left-to-right, right-to-left, bidirectional, or absent) was assessed by color Doppler imaging. When required, supplementary views such as the suprasternal view were used to improve visualization of ductal anatomy. Left pulmonary artery end-diastolic velocity (cm/s) was also recorded. Hemodynamically significant PDA (hsPDA) was defined as a PDA with all of the following echocardiographic findings: (1) left-to-right or bidirectional shunt, (2) a ductal diameter ≥1.5 mm, and (3) a LA-to-aortic root ratio ≥1.5 or a left pulmonary artery end-diastolic velocity ≥20 cm/s, based on previously published criteria. Symptomatic PDA was defined as PDA with left-to-right or bidirectional shunt accompanied by at least two clinical signs (e.g., cardiac murmur, bounding pulse, or pulsus cordis), along with either the requirement for inotropic support or mean blood pressure lower than gestational age (cmH₂O), and worsening respiratory condition (16).

In our clinical practice, prophylactic treatment was not routinely performed; therapeutic intervention was not restricted to infants who fulfilled the criteria for symptomatic or hemodynamically significant PDA. Instead, treatment was initiated based on echocardiographic findings suggestive of a developing hemodynamically significant ductal shunt, even before the PDA became symptomatic or met the criteria for hsPDA, consistent with previously reported management strategies. The timing of PDA closure was defined as the time at which ductal closure was confirmed by echocardiography. Because cranial ultrasonography was performed at discrete time points, the timing of IVH onset was defined as the time at which IVH was first detected. Hemodynamic assessment was performed using echocardiography with an EPIQ 7G, EPIQ Elite, or EPIQ CVx ultrasound system (Philips Healthcare, Andover, MA), equipped with S9-2 or S12-4 transducers.

Cardiovascular support, including dopamine, dobutamine, or corticosteroids, was administered according to clinical judgment based on vital signs and echocardiographic findings. Vasoactive-inotropic score (VIS) was calculated to quantify the degree of cardiovascular support using the following formula: dopamine (µg/kg/min) + dobutamine (µg/kg/min) + 100 × adrenaline (µg/kg/min) + 10 × milrinone (µg/kg/min) + 100 × noradrenaline (µg/kg/min), as previously described (17). VIS was assessed daily during the first three days of life. Maintenance fluid intake included parenteral nutrition, enteral feeding, and maintenance intravenous fluids. Fluid intake and output were expressed as mL/kg/day to account for body weight differences among infants. Net fluid balance was calculated as total fluid intake minus total fluid output and expressed as mL/kg/day. In this study, day 1 was defined as 0–24 h of life, day 2 as 25–48 h, and day 3 as 49–72 h. The timing of indomethacin (IND) or ibuprofen (IBU) administration for symptomatic PDA and follow-up assessments were determined by echocardiograph**y.** Prophylactic IND or IBU was not used. Pharmacologic treatment of PDA was confirmed and followed using echocardiography (16). All included cases were considered to have hemodynamically significant PDA based on clinical and echocardiographic findings. At Kanagawa Children’s Medical Center from 2006 to 2017, IND was administered at 0.1 mg/kg for the first dose, 0.1 mg/kg 24 h later for the second dose, and 0.1 mg/kg 24 h later for the third dose. Since 2018 at the same center, IBU was administered at 2 mg/kg for the first dose, 2 mg/kg 24 h later for the second dose, and 2 mg/kg 24 h later for the third dose. At Kagoshima City Hospital during 2021–2023, IBU was administered at 10 mg/kg for the first dose, followed by 5 mg/kg at 24 and 48 h. Treatment was discontinued if PDA closure was confirmed by echocardiography after any dose.

### Statistical analysis

Continuous variables were expressed as median and interquartile range (IQR) due to non-normal distribution, as assessed by the Shapiro–Wilk test. Categorical variables were presented as counts and percentages. The Mann–Whitney U test was used to compare non-normally distributed continuous variables between the IVH Grade 2–4 group and the non-IVH group. Fisher’s exact test was used for categorical variables. Because all seven IVH events occurred within 12 h after PDA closure, we compared the following parameters during the 0–12-hour post-closure period between groups: ICV pulsation grade, LVDd, ESWS, mVcfc, heart rate (HR), and mean arterial pressure (MAP). Statistical significance was defined as *p* < 0.05, and 95% confidence intervals (CIs) were calculated.

To evaluate the association between hemodynamic parameters and grade 2–4 IVH, as well as their temporal dynamics after PDA closure, generalized linear mixed-effects models (GLMMs) were constructed. For each continuous outcome variable (mVcfc, ESWS, MAP, and HR), separate GLMMs were fitted including time (0, 6, and 12 h after PDA closure), IVH status, and their interaction (time×IVH) as fixed effects, with patient ID included as a random intercept. Estimated marginal means with 95% confidence intervals (CIs) and *p*-values were calculated for each time point and IVH group. The models were implemented using the statsmodels package in Python. Model performance was evaluated by calculating the marginal R², root mean squared error (RMSE), and mean absolute error (MAE). Residual plots and quantile–quantile (Q–Q) plots were examined for diagnostic assessment, and variance inflation factors (VIFs) were calculated to assess collinearity. Statistical significance was defined as *p* < 0.05.

Statistical analyses were conducted using R version 4.3.1 (R Foundation for Statistical Computing, Vienna, Austria) and Python version 3.13 (64-bit).

## Results

A total of 27 infants were admitted during the study period. One infant was excluded from the analysis because IVH had already been diagnosed on the initial cranial ultrasound examination at admission. The final cohort consisted of 26 infants, including 7 who developed grade 2–4 IVH and 19 who did not. The perinatal and clinical characteristics of the infants are summarized in Table 1. PDA closure was confirmed by echocardiography at a median of 40 h after birth (range, 9–64 h). All IVH events were first detected after PDA closure and occurred within 12 h of closure (range, 2–12 h). PDA closure was confirmed within 72 h after birth in all treated cases. Although reopening of the ductus arteriosus was observed in some infants, only one case required subsequent surgical ligation.

**Table 1:** Comparison of perinatal and clinical characteristics between IVH and non-IVH groups.

Variable	IVH group (*n* = 7)	Non-IVH group (*n* = 19)	*p*-value
Perinatal characteristics			
Sex (male)	3 (42.9)	9 (47.4)	1
22 GW	2 (28.6)	2 (10.5)	0.29
Birth weight (median)	520 [437–668]	591 [446–645]	0.28
FGR (10%tile)	1 (14.3)	1 (5.3)	0.47
Twin	1 (14.3)	1 (5.3)	0.47
Primipara	5 (71.4)	14 (73.7)	1
Cesarean delivery	7 (100)	19 (100)	1
MgSO_4_	6 (85.7)	16 (84.2)	1
Ritodrine hydrochloride	6 (85.7)	18 (94.7)	0.47
Antibiotics	3 (42.9)	5 (26.3)	0.64
One course of ACS	4 (57.1)	9 (47.4)	1
PROM	2 (28.6)	12 (63.2)	0.19
CAM stage 2–3	3 (42.9)	8 (42.1)	1
Funisitis stage 2–3	0	8 (42.1)	1
HDP	1 (14.3)	0	0.27
GDM	0	1 (5.3)	1
Abruption	1 (14.3)	1 (5.3)	0.47
Fertility treatment	2 (28.6)	1 (5.3)	0.17
Condition at birth			
APS 1 min	3 [2–5]	2 [1–4]	0.3
APS 5 min	6 [6–7]	6 [4–8]	0.69
UA pH	7.34 [7.12–7.39]	7.36 [7.33–7.39]	0.62
Outcomes and Treatment			
Death	0	1 (5.3)	1
Tension pneumothorax	0	2 (10.5)	1
NEC	1 (14.3)	2 (10.5)	1
FIP	1 (14.3)	2 (10.5)	1
PDA ligation	0	1 (5.3)	1
IND/IBU	5 (71.4)	15 (79.0)	1
HC	5 (71.4)	14 (73.7)	1
Furosemide	7 (100)	13 (68.4)	0.15
Nitroglycerin	3 (42.9)	4 (21.1)	0.34
Fentanyl or Morphine	7 (100)	17 (89.5)	1

Values are presented as median [IQR] or *n* (%). Statistical significance was set at *p* < 0.05. ACS, antenatal corticosteroid, APS, apgar score; CAM, chorioamnionitis; FGR, fetal growth restriction; FIP, focal intestinal perforation; GDM, gestational diabetes mellitus, GW, gestational weeks; HC, hydrocortisone; HDP, hypertensive disorders of pregnancy; IND/IBU, indomethacin/ibuprofen; IQR, interquartile range; IVH, intraventricular hemorrhage; MgSO4, magnesium sulfate; NEC, necrotizing enterocolitis; PDA, patent ductus arteriosus; PROM, premature rupture of membranes; UA pH, umbilical artery pH.

### Clinical management

ACS therapy was administered to 13 neonates (50.0%), and all infants were delivered by cesarean section. Pharmacologic treatment for PDA closure was given to 20 patients (76.9%): 4 received indomethacin and 16 received ibuprofen.

### Univariate and multivariate analyses of risk factors for IVH

Univariate analyses revealed significant between-group differences in post-closure LVDd, post-closure ESWS, post-closure mVcfc, ICV pulsation of at least grade 2, and blood pressure (Tables 2).

**Table 2:** Comparison of parameters within 12 hours after PDA closure (0 vs. 12 Hours).

Variable	IVH group (*n* = 7)	Non-IVH group (*n* = 19)	*p*-value
fluctuation of ICV	0 [−1–2]	0 [−0.3–0.3]	0.85
LVDd (mm)	0.3 [−0.8–1.3]	0.2 [−0.5–0.8]	0.73
ESWS	26.5 [20.2–29.1]	1.8 [−4.8–7.2]	<0.001
mVcfc	−0.68 [−0.70–−0.62]	0.13 [0.0–0.28]	<0.001
HR	17 [3–24]	0 [−7–5]	0.048
MAP	5 [3–6]	0 [−2–2]	0.02

Values are presented as median [IQR]. Statistical significance was set at *p* < 0.05. Comparison of changes in cardiovascular function parameters between IVH (*n* = 7) and control (*n* = 19) groups from PDA closure to 12 h post-closure. The IVH group showed significant increases in ESWS, HR, and MAP, and a significant decrease in mVcfc. ESWS, end-systolic wall stress; fluctuation of ICV, Grade 0–4 fluctuation of internal cerebral vein perfusion waveform; HR, heart rate; IVH, intraventricular hemorrhage; LVDd, left ventricular end-diastolic diameter; MAP, mean arterial pressure; mVcfc, mean velocity of circumferential fiber shortning; PDA, patent ductus arteriosus.

### Generalized linear mixed models (GLMMs)

To investigate the temporal relationship between cardiovascular parameters and IVH following PDA closure, we applied GLMMs for four key variables: ESWS, mVcfc, HR, and MAP. The models showed acceptable fit, with marginal R² values ranging from 0.63 to 0.79. Residual diagnostics showed no major deviations from model assumptions, and multicollinearity was not observed (variance inflation factor < 3 for all predictors).

Compared with infants without IVH, infants with IVH showed significantly higher ESWS, HR, and MAP and significantly lower mVcfc at 6 and 12 h after PDA closure (Table 3).

**Table 3:** Parameter estimates from GLMMs evaluating time×IVH interaction.

Time after PDA closure	Status	ESWS (95% CI)	*p*-value	mVcfc (95% CI)	*p*-value
0 h	Non-IVH	27.2 (24.1–30.3)	<0.001	1.2 (1.1–1.3)	<0.001
	IVH	3.4 (−1.1–7.9)	0.14	−0.05 (−0.17–0.07)	0.45
6 h	Non-IVH	−2.1 (−4.3–0.1)	0.08	0.02 (−0.02–0.06)	0.4
	IVH	8.2 (5.5–10.9)	<0.001	−0.2 (−0.28–−0.12)	<0.001
12 h	Non-IVH	−1.8 (−4.2–0.6)	0.12	−0.01 (−0.06–0.04)	0.7
	IVH	10.1 (7.3–12.9)	<0.001	−0.25 (−0.33–−0.17)	<0.001

Time after PDA closure	Status	HR (95% CI)	*p*-value	MAP (95% CI)	*p*-value
0 h	Non-IVH	144.3 (141.1–147.5)	<0.001	30.4 (28.5–32.3)	<0.001
	IVH	4.3 (−0.7–9.2)	0.09	1.2 (−1.1–3.5)	0.31
6 h	Non-IVH	−2.8 (−5.2–−0.4)	0.02	−0.8 (−1.9–−0.3)	0.14
	IVH	5.7 (1.8–9.6)	0.004	3.5 (1.2–5.8)	0.003
12 h	Non-IVH	0.6 (−1.9–3.1)	0.64	−0.4 (−1.6–0.8)	0.51
	IVH	9.3 (5.1–13.5)	<0.001	4.1 (1.6–6.6)	0.002

Statistical significance was set at *p* < 0.05.Estimated coefficients with 95% confidence intervals and *p*-values from GLMMs assessing the effect of time and IVH status on ESWS, mVcfc, HR, and MAP. Significant interaction terms indicate time-dependent alterations in cardiovascular parameters among infants who developed IVH. CI, confidence interval; ESWS, end-systolic wall stress; HR, heart rate; IVH, intraventricular hemorrhage; MAP, mean arterial pressure; mVcfc, mean velocity of circumferential fiber shortning; PDA, patent ductus arteriosus.

ESWS increased significantly in the IVH group at 6 h (estimate, 8.2; 95% CI, 5.5–10.9; *p* < 0.001) and 12 h (estimate, 10.1; 95% CI, 7.3–12.9; *p* < 0.001). mVcfc decreased significantly at 6 h (estimate, −0.20; 95% CI, −0.28 to −0.12; *p* < 0.001) and 12 h (estimate, −0.25; 95% CI, −0.33 to −0.17; *p* < 0.001). HR increased at 6 h (estimate, 5.7; 95% CI, 1.8–9.6; *p* = 0.004) and 12 h (estimate, 9.3; 95% CI, 5.1–13.5; *p* < 0.001). MAP increased at 6 h (estimate, 3.5; 95% CI, 1.2–5.8; *p* = 0.003) and 12 h (estimate, 4.1; 95% CI, 1.6–6.6; *p* = 0.002).

### Between-group comparison of changes during the first 12 h

Wilcoxon tests comparing changes from PDA closure to 12 h after closure confirmed significant between-group differences in ESWS, mVcfc, HR, and MAP (all *p* < 0.05). No significant between-group differences were observed in ICV pulsation grade or LVDd changes (Table 2). These findings suggest that IVH occurring within 12 h after PDA closure is associated with increased afterload, impaired myocardial contractility, and hemodynamic instability.

VIS values were significantly higher in the IVH group than in the non-IVH group during the first three days of life (Day 1: 8 [6–10] vs. 2 [0–5], *p* = 0.004; Day 2: 8 [8–10] vs. 0 [0–6], *p* = 0.002; Day 3: 10 [10–12] vs. 1.5 [0–8], *p* = 0.003). Net fluid balance was significantly higher in the IVH group on days 2 and 3 (Table 4).

**Table 4:** Cardiovascular support (VIS) and fluid balance during the first 3 days of life.

Variable	IVH group (*n* = 7)	Non-IVH group (*n* = 19)	*p*-value
IN (day 1)	115.0 [81.7–172.0]	87.9 [83.0–106.1]	0.31
IN (day 2)	120.6 [102.2–124.6]	95.7 [85.5–107.0]	0.01
IN (day 3)	119.7 [99.6–133.5]	103 [90.4–108.1]	0.13
OUT (day 1)	78.1 [48.7–87.1]	52.8 [31.2–73.9]	0.053
OUT (day 2)	85.6 [52.3–128.6]	83.9 [67.7–104.3]	0.79
OUT (day 3)	55.0 [44.0–89.1]	55.0 [60.0–117.3]	0.035
NFB (day 1)	80.2 [38.2–112.7]	57.0 [28.0–58.8]	0.18
NFB (day 2)	63.3 [24.4–71.9]	8.3 [−5.1–26.8]	0.01
NFB (day 3)	57.8 [13.6–78.5]	9.0 [−22.9–39.3]	0.04
VIS (day 1)	8 [6–10]	2 [0–5]	0.004
VIS (day 2)	8 [8–10]	2 [0–6]	0.002
VIS (day 3)	10 [10–12]	2 [0–8]	0.003

Values are presented as median [IQR]. Statistical significance was set at *p* < 0.05. IQR, interquartile range; IVH, intraventricular hemorrhage; NFB, net fluid balance; VIS, vasoactive-inotropic score.

### Model performance and diagnostics

For ESWS, the model showed a marginal R² of 0.71 [95% confidence interval (CI): 0.65–0.76], with a RMSE of 10.47 (95% CI: 9.2–11.8) and a MAE of 8.85 (95% CI: 7.6–10.1). For mVcfc, the marginal R² was 0.74 (95% CI: 0.68–0.79), the RMSE was 0.21 (95% CI: 0.17–0.26), and the MAE was 0.19 (95% CI: 0.15–0.23). For HR, the marginal R² was 0.63 (95% CI: 0.55–0.71), the RMSE was 6.11 (95% CI: 5.1–7.2), and the MAE was 4.15 (95% CI: 3.4–4.9). For MAP, the marginal R² was 0.79 (95% CI: 0.73–0.84), the RMSE was 7.17 (95% CI: 6.0–8.3), and the MAE was 6.34 (95% CI: 5.3–7.2).

## Discussion

In this study, extremely preterm infants who developed IVH within 12 h after PDA closure exhibited distinct early hemodynamic alterations compared with those who did not. Using GLMMs, divergence in cardiovascular parameters became apparent as early as 6 h after closure. Specifically, infants with IVH showed significant increases in ESWS, MAP, and HR, along with a significant decrease in mVcfc. These findings indicate that the immediate post-closure period may represent a critical window of hemodynamic vulnerability in infants born at 22–23 weeks’ gestation. However, because blood pressure also rises during this period, the increase in afterload cannot be attributed solely to ductal closure (1). Some infants may show reduced ventricular size after closure and thus lower wall stress, while in others—without such remodeling—ESWS increases, potentially predisposing to IVH (2, 3). These findings suggest that maladaptive cardiovascular responses after PDA closure may contribute to the development of IVH rather than representing normal transitional physiology alone. However, this interpretation should be considered hypothesis-generating rather than evidence of causality. Unlike arterial Doppler indices, ICV pulsation may reflect cerebral venous congestion and central venous pressure, which could be particularly relevant to the pathophysiology of IVH. The divergence in cardiovascular parameters between groups suggests that these changes are not simply attributable to postnatal maturation. In addition, infants who developed IVH required significantly higher vasoactive support, as reflected by elevated VIS values during the first three days of life. This finding is consistent with greater circulatory instability in the IVH group; however, whether increased vasoactive support contributed to IVH development or merely reflected underlying hemodynamic compromise remains unclear. Post-closure echocardiographic findings revealed increased left ventricular afterload, decreased wall motion, and elevated HR compared to pre-closure assessments. In addition, higher fluid intake and lower output on day 2 in the IVH group may suggest fluid retention, which could have increased cardiac loading conditions. Consistent with this, net fluid balance was higher in the IVH group, particularly during the early postnatal period, suggesting increased intravascular volume and cardiac loading conditions. Taken together, these observations suggest that although left ventricular preload may decrease following PDA closure due to cessation of shunt flow, the abrupt increase in afterload may impair contractility. In response, the heart attempts to compensate through tachycardia; however, this adaptive response may be inadequate, leading to left ventricular failure and secondary impairment of right ventricular function. One possible consequence is elevation of central venous pressure with impaired cerebral venous return, which may contribute to IVH. However, direct assessment of preload-related parameters was not performed in this study. Because this was a retrospective observational study, this proposed mechanism should be regarded as hypothesis-generating rather than causal. Our results suggest that careful circulatory monitoring and individualized fluid management immediately after PDA closure may be critical in this population, although causal relationships cannot be established in this retrospective study. Whether early interventions such as diuretics or vasodilators improve outcomes warrants further investigation. It should also be noted that hemodynamic findings may have been influenced by several confounding factors, including vasoactive support, pharmacological treatment for PDA, and fluid status.

Previous studies have reported that vasodilator therapy can reduce ESWS and thereby decrease left ventricular afterload. Nitroglycerin, in particular, has been shown to lower blood pressure, reduce ESWS, and improve mVcfc, supporting its potential utility in managing cardiovascular stress following PDA closure. In addition, phosphodiesterase type 3 (PDE3) inhibitors such as milrinone have been reported to exert vasodilatory effects not only systemically but also on the ductus arteriosus itself (18, 19). Therefore, while PDE3 inhibitors may improve myocardial performance and reduce afterload, their ductal vasodilatory properties raise concerns regarding maintenance or re-opening of ductal patency.

However, caution is warranted when using vasodilators such as nitroglycerin and phosphodiesterase (PDE) inhibitors, including milrinone. These agents, while effective in improving myocardial performance and reducing afterload, may also promote ductal patency or even contribute to re-opening of the ductus arteriosus. In the early post-closure phase, particularly in extremely preterm infants, the risk of PDA re-canalization should be carefully weighed against the potential cardiovascular benefits (6). Close echocardiographic monitoring and individualized hemodynamic assessment are essential when considering their use. This study has several limitations. First, the sample size was small, reflecting the rarity of infants born at 22–23 weeks’ gestation, which may limit statistical power and generalizability. Second, the retrospective design introduces the possibility of selection bias and unmeasured confounding. Third, echocardiographic assessments were performed by multiple clinicians, which may introduce interobserver variability. Advanced echocardiographic techniques such as tissue Doppler imaging or strain analysis were not applied in this study, as their feasibility and reliability in extremely preterm infants at 22–23 weeks’ gestation remain limited. Fourth, detailed assessment of right ventricular function, pulmonary hemodynamics, and systemic blood flow (e.g., superior vena cava flow) was not systematically performed, which may limit interpretation of the underlying hemodynamic mechanisms. Finally, management strategies, including fluid administration and cardiovascular support, were not standardized across centers. Prospective multicenter studies are needed to validate these findings and determine whether targeted hemodynamic interventions during the immediate post-closure period can reduce IVH risk.

## Conclusion

In infants born at 22–23 weeks’ gestation, hemodynamic instability emerging within 12 h after ductal closure—characterized by increased ESWS, MAP, and HR together with decreased mVcfc—was associated with subsequent grade 2–4 intraventricular hemorrhage. Recognition of this early post-closure pattern may support risk stratification and more targeted circulatory monitoring in this highly vulnerable population.

## Data Availability

The original contributions presented in the study are included in the article/supplementary material, further inquiries can be directed to the corresponding author.
